# Change in Surface Roughness on the Inner and Outer Surfaces of the Microtube during Hollow Sinking

**DOI:** 10.3390/ma17174320

**Published:** 2024-08-30

**Authors:** Hayate Sakaguchi, Takuma Kishimoto, Saki Suematsu, Kenichi Tashima, Koichi Kano, Satoshi Kajino, Shiori Gondo, Shinsuke Suzuki

**Affiliations:** 1 Department of Materials Science, Graduate School of Fundamental Science and Engineering, Waseda University, 3-4-1 Okubo, Shinjuku 169-8555, Japan; suzuki-s@waseda.jp; 2 Department of Applied Mechanics and Aerospace Engineering, Faculty of Fundamental Science and Engineering, Waseda University, 3-4-1 Okubo, Shinjuku 169-8555, Japan; ghkikuiibb@ruri.waseda.jp; 3 Department of Physical Science and Technology, Nagoya Institute of Technology, Gokiso-cho, Showa-ku, Nagoya 466-8555, Japan; takuma-k@iis.u-tokyo.ac.jp; 4 Department of Applied Mechanics and Aerospace Engineering, Graduate School of Fundamental Science and Engineering, Waseda University, 3-4-1 Okubo, Shinjuku 169-8555, Japan; suematsu_s@asagi.waseda.jp; 5 Factory-Automation Electronics Inc., 1-6-14 Higashi-yodogawa, Osaka 533-0033, Japan; k-tashima@fae.jp; 6 National Institute of Advanced Industrial Science and Technology (AIST), Tsukuba East, 1-2-1 Namiki, Tsukuba 305-8564, Japan; kajino-satoshi@aist.go.jp (S.K.); shiori-gondo@aist.go.jp (S.G.); 7 Kagami Memorial Research Institute of Materials Science and Technology, Waseda University, 2-8-26 Nishi-waseda, Shinjuku 169-0051, Japan

**Keywords:** stainless steel, microtube, tube drawing, hollow sinking, uniaxial tensile deformation, surface roughness

## Abstract

Hollow sinking experiments and tensile tests were conducted to clarify the evolution of surface roughness during hollow sinking. Stainless steel tubes (outer diameter: 1.5 mm; wall thickness: 0.045 mm) featuring a single grain spanning the wall thickness achieved via annealing as the starting material. The tube was drawn without an internal tool using a draw bench by controlling the tube drawing speed ratio of the die entrance and exit sides. The surface roughnesses of the inner and outer surfaces at the die entrance and exit sides of the drawn tube were compared with the surface roughnesses of the inner and outer surfaces under the uniaxial tensile deformation of the starting material. As a result, two major findings were revealed; the surface roughness formation behavior during the hollow sinking; the uniaxial tensile deformation exhibits a tube on both sides of the entrance and the exit of a die. Former uniaxial tensile deformation forms surface roughness of the tube at the die-entrance-side. However, hollow sinking reduces the roughness. The tube keeps its small roughness even though it is applied the later uniaxial tensile deformation behind the die exit. Furthermore, the conventional formula to predict the surface roughness of a metal sheet caused by the uniaxial tensile deformation can predict the surface roughness of a tube in the hollow sinking. At both die entrance and exit sides, the roughness of the inner surface was larger than that of the outer surface at the die entrance and exit side. The outer surface of the tube contacts the inside of a die when the tube passes through the die. The height of the convex parts decreased at that moment. Hollow sinking suppressed the increase in surface roughness of the inner surface as the outer surface was smoothed in the die. However, due to the formation of surface roughness after leaving the die, there is an overall increasing trend in inner surface roughness.

## 1. Introduction

Hollow sinking is one of the most suitable drawing processes for fabrication of thin-walled metal microtubes. The hollow nature of metal microtubes allows them to be used for the flow of liquids such as chemicals or gases. Therefore, they are widely applied in the medical, chemical, and biotechnological fields and are in high demand [1]. Especially in the medical field, they are used for painless injection needles and medical stents [2,3,4]. The outer diameter of the painless injection needles should be 0.1 mm or less [5]. Medical stents must have a smooth inner surface because of the risk of blood vessel re-occlusion. The large surface roughness of the stent leads to a faster corrosion rate in the body [6]. Medical stents need to remain in the body for three to six months. Therefore, the arithmetic mean line roughness *R*_a_ of the stent must be at least less than *R*_a_ = 0.5 μm [7,8]. Therefore, further miniaturization of the outer diameter and low surface roughness of metal microtubes is required.

The main conventional fabrication methods of metal microtubes are drawing, bending, and spinning. In particular, drawing is considered the most suitable process for manufacturing metal microtubes in terms of precision and productivity [1]. In the current drawing process, the hollow shape of metal microtubes makes it difficult to insert an internal tool as the diameter of the tubes is fine. Therefore, hollow sinking, which does not require an internal tool, is suitable for reducing the diameters of metal microtubes. However, because the inner surface does not contact the die, the wall thickness tends to increase, and the surface becomes rough [2]. Therefore, in previous study, we established a theory of thickness control by precisely controlling the tube drawing speed ratio based on the constant volume law [9]. Furthermore, it has been clarified that the excessive thinning of outer diameter is caused by the excessive elastic strain associated with plastic anisotropy. Excessive thinning is caused by uniaxial tensile deformation, which begins at the approaching part of the die. In this research, the die-entrance-side is defined as before the metal microtubes contact with the die. Conversely, the die-exit-side is defined as after the metal microtubes pass through the die approach. The back and front tensions acting on the tubes at die entrance and exit sides are lower than the yield points of the bulk material. However, micro-effects have been shown to cause localized yielding and excessive elastic strain [10]. In thin-walled materials, when there are only a few grains across the thickness, the material yields lower stresses than bulk material, and the slope of the elastic region is smaller. In addition, the elastic recovery is greater than that of the bulk material, resulting in excessive elastic strain. We have shown that excessive elastic strain causes excessive thinning. Our studies enable high-precision control of these dimensions. However, the mechanism of surface roughening has not yet been clarified for the issue of surface roughness.

The previous studies have found that in the hollow sinking of metal microtubes, not only the inner surface but also the outer surface was roughened owing to excessive thinning of the outer diameter [11]. The surface roughness becomes non-negligible for metal microtubes with dimensions of 1 mm or less. One of the grain orientations suppress the inner surface roughness has been found as the orientation {102}, which is normal to the inner surface [12]. However, it remains to be clarified whether the three-dimensional deformation of the crystal grains is responsible for the formation of concave and convex structures, as is the case with sheet materials. In the case of metal microtubes, the outer surface is conventionally considered smooth owing to contact with the die; therefore, the relative positions of the outer and inner surface roughnesses have not been examined. Therefore, it is necessary to first clarify the relationship between the outer and inner surface roughnesses.

The number of grains across the wall thickness direction became extremely small, and the ratio of the grain diameter to the wall thickness increased as the diameter of the metal microtubes decreased [12]. Therefore, it is considered that grain rotation and slip deformation are deeply involved in the formation of surface roughness in drawings, as is the case with the inhomogeneous formation in sheet materials with a small number of grains [13,14,15,16,17,18,19,20,21,22].

In hollow sinking, the outer surface is contact with the die, whereas the inner surface is free. It is thought that, as in the case of sheet materials, the deformation and rotation of crystal grains cause convex and concave parts (referred to as unevenness in this paper) in the drawn tube materials. Therefore, in the die, we hypothesized that the inner surface will approach smoothness as the outer surface is smoothed when the number of grains in the direction of the wall thickness is extremely small.

For sheet metals, it has been shown that the arithmetic mean line roughness *R*_a_ increases almost proportionally with the plastic strain, regardless of tension or compression. Furthermore, it has become clear that the local deformation of grains in the surface layer causes surface roughness. Surface roughening is caused by inhomogeneous deformation owing to the differences in deformation resistance between grains [13,14,15,16,17,18,19,20,21,22]. It has been found that the increase in surface roughness on the inner surface was greater than that of drawing with an inner tool during hollow sinking [2]. The surface roughness of the tube materials is difficult to measure because of the curved nature of their inner and outer surfaces. Only few studies about surface roughness of tube have been conducted [23,24,25,26,27,28]. Consequently, it is inferred that the surface roughness depends on the grain size. It has also been suggested that the surface properties are modified after plastic deformation when the Taylor factor is small [23]. The roughness increment decreased exponentially with increasing strain, and the surface roughness increased with strain at values below 0.067 and then decreased slightly. In-situ SEM observations revealed that the surface roughness is caused by inhomogeneous deformation between and within the grains. The slip of adjacent grains dispersed the concentrated deformation. With increasing strain, the deformation became more nonuniform. When the strain exceeds a critical value, the deformation becomes more homogeneous as more local deformation bands of the slip band participate in the deformation, resulting in a slight decrease in the surface roughness [26]. Loginov et al. studied the roughness before and after drawing copper tubes by measuring the inner surface roughness, *R*_a_, corresponding to each drawing strain [27]. They speculated that the deterioration of the surface conditions was related to the rotation of crystal grains near the inner surface layer. It was also found that the ratio of the increase in the roughness parameter was the greatest after one pass [27].

Therefore, this study aimed to clarify the change in surface roughness during the hollow sinking of metal microtubes. The surface roughness on the die entrance and exit sides is formed by uniaxial tensile deformation starting at the die approach. The relationship between the surface roughness of the inner and outer surfaces of the drawn tube at the same position and the unevenness of the inner and outer surfaces of the die was examined. The surface roughnesses on the inner and outer surfaces of the die entrance and exit sides were compared with that on the inner and outer surfaces during uniaxial tensile deformation.

## 2. Experimental Procedure

### 2.1. Materials and Hollow Sinking

Stainless steel tubes (SUS304) with an outer diameter of *D*_0_ = 1.5 mm and a wall thickness of *t*_0_ = 0.045 mm, which were fabricated by plug drawing, were used as the starting material, which were fabricated by Fuji Seiko Co., Ltd. (Shizuoka, Japan). The starting materials were annealed to obtain tubes containing one grain along the wall thickness direction. Annealing was performed at 1273 K for 60 min by Tamayakin Co., Ltd. (Tokyo, Japan) so that the grain diameter should be coarsened to more than 0.045 mm. The heat treatment was applied to enhance the correlation of surface roughness between inner and outer surfaces and link the surface roughness to the deformation of each grain in the direction of the wall thickness as one grain. The starting material used in this study was the same as in the our previous research [12]. Therefore, the chemical compositions are also the same as the reference [12]. Table 1 lists the chemical compositions of the specimens. The chemical composition of the stainless steel tubes was within the standard range according to the Japanese Industrial Standard JIS-G4305 [29].

**Figure 1:**
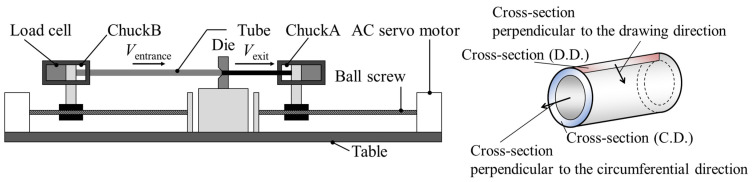
Schematic illustration of hollow sinking controlled drawing speed in the exit side *V*_exit_ and *V*_entrance_ using a speed-ratio draw bench (Factory-Automation Electronics Inc.).

The starting materials were drawn in a single pass by hollow sinking using a speed-ratio draw bench (Factory-Automation Electronics Inc., Osaka, Japan). The tubes were drawn with high-precision control of the die entrance and exit side speeds. Bright Lube (Sanpo Chemical Works Co., Ltd., Tokyo, Japan, 124 MS) was used as the liquid lubricant. The equipment used is the same as in previous research [12]. Figure 1 shows a schematic illustration of the drawing bench. A chuck connected to a ball screw was used to grip the tube at the die entrance and exit sides. The tube drawing speed was controlled by precisely controlling the moving speed of each chuck using the rotational speed of the AC servomotors at the die entrance and exit sides. The drawing speed in the exit side *V*_exit_ and the speed in the entrance side *V*_entrance_ were set to 5.0 mm/s and 4.4 mm/s so that the drawing speed ratio *β* (=die exit speed *V*_exit_/die entrance speed *V*_entrance_) should be 1.14, which is the condition of the wall thickness reduction [9]. The drawn tube was not coiled but was collected while maintaining straightness to suppress tube deformation. The drawing was interrupted to observe the halfway-drawn tubes. At the end of the drawing, the drawn tube was stopped halfway and drawn out on the opposite side of the drawing direction to obtain the tube remaining inside the die. The hollow sinking was terminated when the chuck on the die exit side reached the end of the ball screw.

### 2.2. Uniaxial Tensile Deformation of Starting Material

Tensile tests were performed to measure the surface roughness of the tube caused by uniaxial tensile deformation. To compare with the roughness of the drawn tube, uniaxial tensile deformation of the starting material was conducted using a universal testing machine (Shimadzu, Autograph AG-25TB, Kyoto, Japan) (hereinafter called “tensile-deformed tubes”). The initial distance between the chucks was set to 100 mm, a 5 kN fine wire gripper (Shimadzu, 343-07529-01, Kyoto, Japan) was used to chuck the tube, and the test speeds were 5 mm/min and 300 mm/min which are called in this paper low speed test and high speed test, respectively. The test force was measured using a 5 kN load cell (Shimadzu, SLBL-5kN, Kyoto, Japan). The displacement of the cross-head was measured as the tube stroke. The stroke was set to either 8 mm or 14 mm to be the same as the strain in the drawing direction of the elongated tube described above.

**Figure 2:**
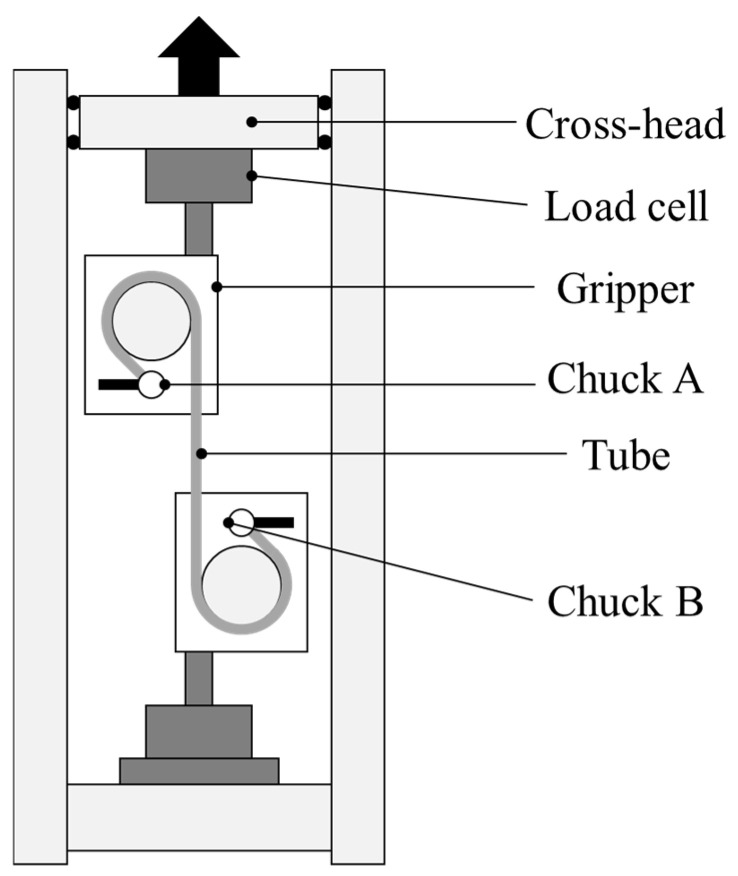
Schematic illustration of starting material setting in the universal testing machine for a tensile test [12].

A previous study of our research group revealed that the spring-back of thin-walled metal microtubes with a very small number of grains across the wall thickness was not linear when unloaded after uniaxial tensile deformation [10]. Loading–unloading tests were also conducted, in which a load was applied up to a certain value and then unloaded to estimate the plastic strain at the maximum strain when given load. The load was set to 45–60 N because the load at the die entrance side was 50–55 N, as described in Section 3.1. Results of loading–unloading test and drawing tests. The load was controlled using a PID controller. After the load reached the maximum value, it was gradually decreased by stopping the hydraulic pump, which controlled the displacement. The amount of plastic strain in the drawing direction was 0.08 and 0.14, with an initial length of 100 mm. These values were according to the results of the inner and outer diameter measurements described below, based on the constant volume law described in 2.4 Observation of cross-section. A schematic illustration of the sample setting for the universal testing machine is shown in Figure 2. The universal testing machine used in this study was the same with the previous research [12].

### 2.3. Observation of Inner and Outer Surfaces

**Figure 3:**
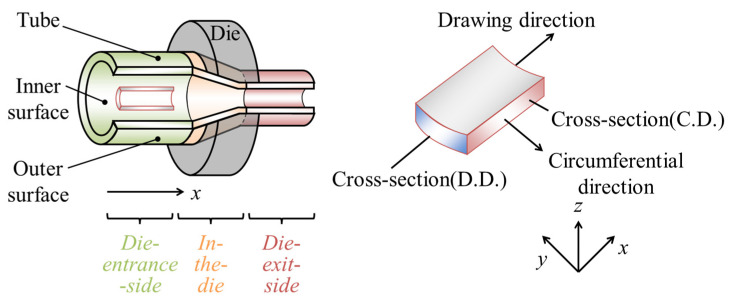
Schematic illustration of a hollow sinking with partial cross-sections of the tube, measurement points, and a sample cut from a tube.

A confocal laser microscope (KEYENCE Co., VK9510, Osaka, Japan) was used to obtain images and three-dimensional profiles of the inner and outer surfaces of the starting material, halfway-drawn tubes, and tensile-deformed tubes. The method of obtaining halfway-drawn tubes is described in detail in Section 2.1. For the halfway-drawn tube, the die entrance side, die, and die exit side were observed using a laser microscope to observe the change in unevenness during drawing. The observation areas of the halfway-drawn tube were classified into “die-entrance-side”, “in-the-die”, and “die-exit-side” in length (drawing) direction. A schematic of the measurement points is shown in Figure 3. The scanning length of measurement area is 0.278 × 1024 = 286.7 μm in the vertical direction and 0.278 × 768 = 213.5 μm in the horizontal direction. The number 0.278 μm indicates the resolution at 50× eyepiece magnification. The sampling positions were taken at the center of the tube in the drawing (longitudinal) and circumferential directions. During the observation of the surface, the same position was measured using a scale displaying the machine screen at the inner and outer surfaces of the tubes using an incision on the material as a mark. To evaluate surface unevenness, a cutoff was not used in this study to remove surface undulation and noise but rather to remove anomalous height values due to surface deposits and noise generated when converting laser light intensity to height.

The height distribution of surface unevenness was obtained using the following procedure: First, *z* = 0 was set as the lowest point in the measurement field of view with the laser emitted from the microscope irradiation in the *z*-axis direction. Then the *z*-coordinate of each pixel in the measurement field of view was acquired. Because the tube had a curved surface, the acquired coordinates were corrected for the curved surface using MATLAB^TM^ (Mathworks, R2021b, Natick, MA, USA) and Equation (1), which represents the second-order correction in the *y*-direction and the first-order correction in the *x*-direction. The subscripts *i* and *j* denote the number of pixels in the *x*-direction and *y*-direction, respectively. Coefficients *a_mn_* were calculated by fitting. The symbols *m* and *n* correspond to the orders of *x* and *y*. The deviation from the mean (reference plane *z* = *z*_0_) in the corrected *z*-coordinates is defined as height *h*. The relative frequency, *f*, which is a quantitative index of height, was calculated as the ratio of the frequency of height, *h*, to the number of pixels in the observed area. The height *h* is given by Equation (2).

*z*_0*ij*_ = *a*_02_*y_j_*^2^ + *a*_11_*x_i_y_j_* + *a*_01_*y_j_* + *a*_10_*x_i_* + *a*_00_ (1)

*h_ij_* = *z_ij_* − *z*_0*ij*_ (2)

The arithmetic mean surface roughness *R*_a_ was calculated by Equation (3) using the height *h_ij_*. The symbols *X* and *Y* represent the number of pixels in the *x*-direction and *y*-direction, respectively. There were 768 pixels in the *x*-direction and 1024 pixels in the *y*-direction.

(3) $$R_{a}=\frac{1}{n}\sum_{j=1}^{Y}\sum_{i=1}^{X}\left|h_{ij}\right|$$

Fitting was performed on the obtained relative frequency *f* distributions of the heights using MATLAB^TM^. Only the starting material was fitted with a Gaussian function with one peak, whereas the halfway-drawn tube at the die entrance, die, and die exit sides were fitted with the sum of Gaussians with two peaks.

Furthermore, in this study, grain boundaries were observed in microphotographs of the inner and outer surfaces. Grain boundaries were at dark continuous line on the microphotograph using MATLAB^TM^ (Mathworks, R2023b, Natick, MA, USA). Each grain was binarized and the areas of grains were calculated by MATLAB^TM^, and the grain size was calculated as the equivalent circular diameter (ECD). The product of ECD and the area fraction was calculated for each grain, the sum of which was defined as the average ECD. From the calculation results, the grain sizes of the outer and inner surfaces were 48.9 μm and 43.0 μm, respectively. There were approximately 30 and 39 grains in the inner surface and outer surface of images, respectively. In addition, the distribution of ECD was close to a normal distribution. Therefore, the 95% confidence interval of the normal distribution was used in the calculations.

### 2.4. Observation of Cross-Section

To obtain the inner and outer diameters of the starting material, halfway-drawn tubes, and uniaxial tensile-deformed tubes, circumferential cross-sections were observed. Images were obtained using a three-dimensional (3D) microscope (KEYENCE Co., VHX-5000, Osaka, Japan) and analyzed to calculate the outer diameters using MATLAB^TM^. The numbers of pixels in the inner and outer diameters were converted into dimensions using the number of pixels in the scale-obtained images. Based on the calculated inner and outer diameters, the amount of strain in the drawing direction was calculated using the constant volume law.

To observe the correspondence between the unevenness of the inner and outer surfaces, a cross-section of the wall was observed. The specimens were resin-filled and polished to expose the cross-section of the wall so that its plane passed through the center of the tube. The inner and outer surfaces were plated to suppress the deformation during polishing. The cross-sectional photographs of the resin-filled tubes were obtained using a 3D microscope. The coordinates of the top and bottom edges were read using MATLAB^TM^ (Mathworks, R2021b, Natick, MA, USA), and the unevenness of the inner and outer surfaces was calculated using the acquired cross-sectional photographs.

Electron backscatter diffraction (EBSD) (TSL solutions Co., Ltd., OIM7, Kanagawa, Japan) was conducted to observe the grains on the cross-sections perpendicular to the circumferential direction (C.D.) and the drawing direction (D.D.). Inverse pole figure (IPF) maps of the circumferential and thickness cross-sections were obtained by EBSD. The ECD of each grain in circumferential and wall thickness directions were calculated by OIM analysis (EDAX Inc., Version 7.3.1, Pleasanton, CA, USA). The grain sizes of the circumferential and wall thickness directions were 41.3 μm and 33.3 μm, respectively. There were approximately 30 and 1.6 × 10^5^ grains in the normal to cross-section of the circumferential direction and the wall thickness direction, respectively. The half-way grain at the image boundary was also calculated as one grain, but they were omitted by the following process. The distribution of ECD was close to a normal distribution. Therefore, the 95% confidence interval of the normal distribution was used in the calculations.

## 3. Results

### 3.1. Loading–Unloading Tensile Tests and Hollow Sinking Experiments

**Figure 4:**
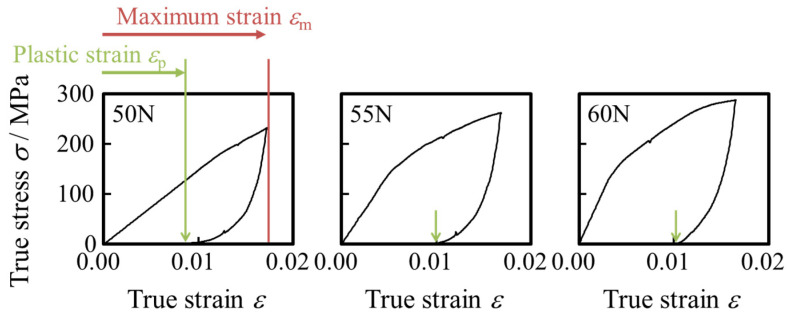
Results of the loading–unloading test. The horizontal axis represents true strain *ε*, and the vertical axis represents true stress *σ*. The maximum loads are shown in the top left-hand corner of the figures, respectively. The arrows in the figure indicate the strain after unloading, which is the plastic strain *ε*_p_.

Figure 4 shows the results of the loading–unloading tensile tests of the starting materials under maximum loads of 50, 55, and 60 N. The true strain after unloading in each loading–unloading test was about 0.01. The plastic strain increased with increasing maximum load. Plastic strain is the true strain remaining after unloading. The load did not decrease linearly after unloading. Furthermore, the slope of the line in the linear region ranged from 14.8 to 41.3 GPa, which is much smaller than Young’s modulus of stainless steel (210 GPa) in the bulk material. The results of the loading–unloading tensile tests indicate that the micro-effect occurs in the starting materials, as in our previous research [8].

**Figure 5:**
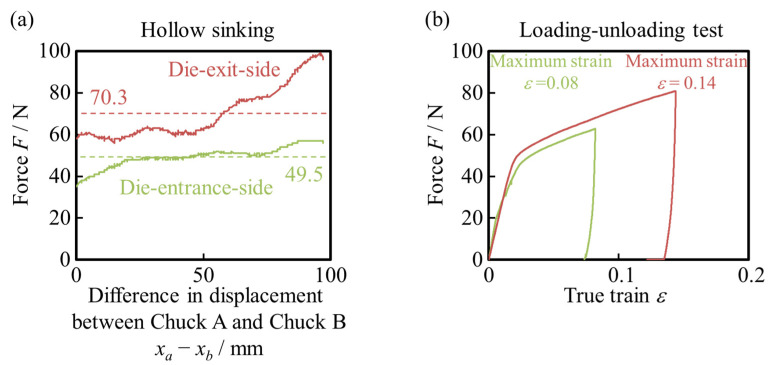
(**a**) Changes in drawing force during hollow sinking. The dotted line indicates the average value during the stable state. (**b**) Results of load–unloading uniaxial tensile test. The strokes were the same value on the die-entrance-side and die-exit-side, respectively.

Figure 5 shows the load transition during hollow sinking and loading–unloading uniaxial tensile tests of the starting material. The loads at the die-entrance-side and the die-exit-side correspond to the back tension and front tension of hollow sinking, respectively. The front tension minus the back tension is the drawing force during hollow sinking. The maximum strains in the tensile tests were set to the same values as the strains in the die-entrance-side and die-exit-side, respectively. Therefore, the strokes of the loading–unloading uniaxial tensile test can be considered the same for the die-entrance-side and die-exit-side, respectively. The amount of strain loaded by the uniaxial tensile test was consistent with the strain during hollow sinking under the conditions described in Section 2.2 and Section 2.4. Therefore, the longitudinal strain in the loading–unloading uniaxial tensile test was equivalent to the strain in the drawing direction of the hollow sinking.

**Figure 6:**
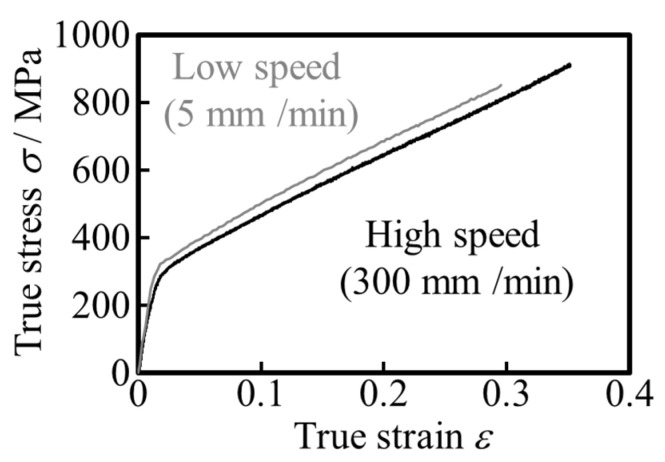
Uniaxial tensile tests at different test speeds for strain rate dependence of starting material (stainless steel tube).

Additional uniaxial tensile tests of different test speeds were conducted to check the strain rate dependence. Figure 6 shows the strain-rate dependence of the deformation of the starting material used in this study. According to Figure 6, stress of the high speed test is about 50 MPa higher than that of low speed test. This result suggests that the slight strain rate dependency exists in specimen used in this study. Therefore, the load of hollow sinking is smaller than that of loading unloading test.

### 3.2. Observation Results of Unevenness on Inner and Outer Surfaces

**Figure 7:**
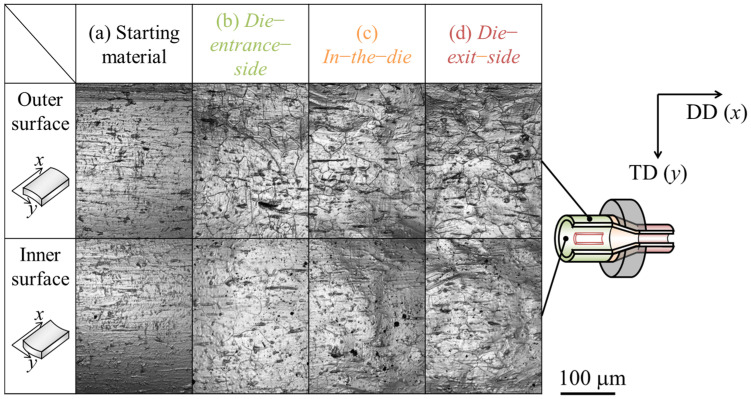
Microphotographs of inner and outer surfaces of the (**a**) starting material, (**b**) die-entrance-side, (**c**) in-the-die, and (**d**) die-exit-side of the halfway-drawn tube. The symbols DD and TD indicate the drawing direction and transversal direction, respectively.

**Figure 8:**
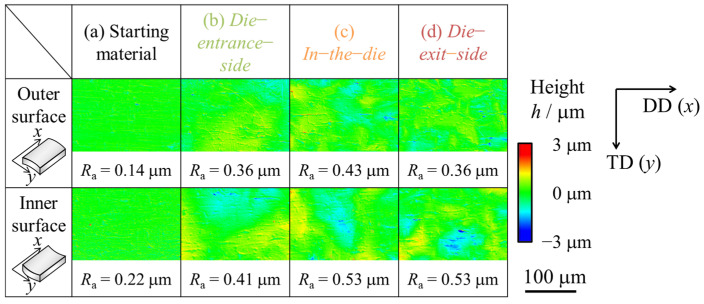
Height distribution recorded by a laser microscope of the inner and outer surfaces of the (**a**) starting material, (**b**) die-entrance-side, (**c**) in-the-die, and (**d**) die-exit-side of the halfway-drawn tube. Height *h* represents the deviation from the mean value. The arithmetic mean surface roughness, *R*_a_, represents the average of the absolute value of height, *h*.

Figure 7 and Figure 8 show the microphotographs, height distributions, and 3D profiles of the inner and outer surfaces of the starting material and drawn tubes. The results for both the inner and outer surfaces are presented for the die-entrance-side, in-the-die, and die-exit-side. It was revealed that unevenness formed from the die-entrance-side, and the inner and outer surfaces were roughened. This was because the *R*_a_ had increased twice at the die-entrance-side. The absolute height of the convex structure formed on the die-entrance-side was larger than that of starting material. According to Figure 8, there is a trend of decreasing roughness on the outer surface of the tube, while the roughness on the inner surface remains unchanged at die-exit-side and die. In this study, it was difficult to clarify a direct factor for inner surface roughness suppression when outer surface roughness decreased. Figure 7 shows that there are lines parallel to the drawing direction on the inner and outer surfaces. Traces of contact were found between the die-entrance-side and in-the-die. We found that the *R*_a_ of the inner surface decreased with a decrease in the *R*_a_ of the outer surface.

**Figure 9:**
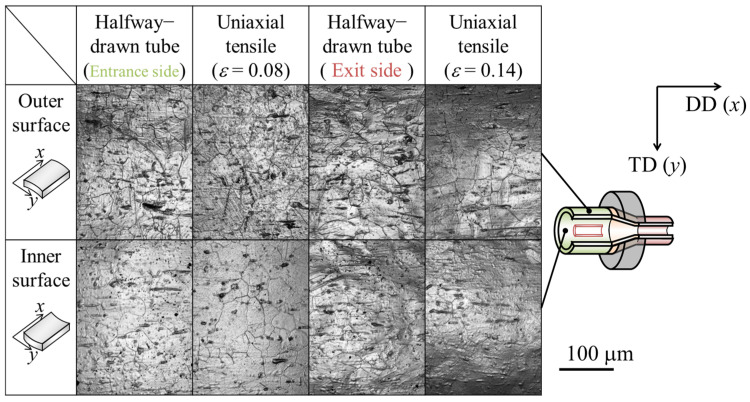
Microphotographs of inner and outer surfaces of the die-entrance-side, uniaxial tensile- deformed tube with a strain of 8%, die-exit-side of the drawn tube, and uniaxial tensile deformed tube with a strain of 14%, respectively.

**Figure 10:**
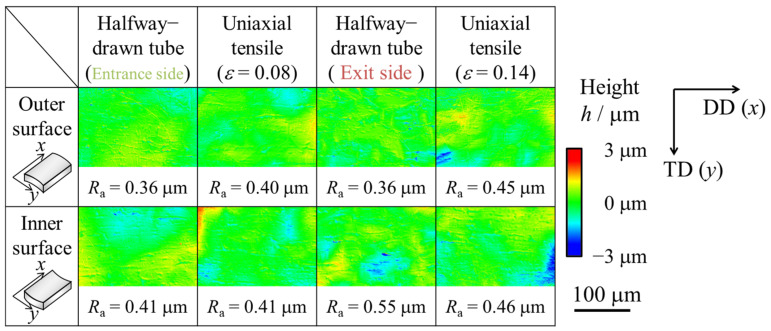
Height distribution of inner and outer surfaces of die-entrance-side, uniaxial tensile deformed tube with a strain of 8%, die-exit-side of the drawn tube, and uniaxial tensile deformed tube with a strain of 14%, respectively. Height *h* represents the deviation from the mean value. The arithmetic mean surface roughness *R*_a_ represents the average of the absolute value of height *h*.

Microphotographs, height distributions, and 3D maps of the inner and outer surfaces of the uniaxial tensile-deformed are shown in Figure 9 and Figure 10, respectively. The samples after uniaxial tensile deformation had the same elongation ratio as the halfway-drawn tube and they were elongated 1.08 times during the die entrance side and 1.14 times during the die exit side. These values were based on the results of the inner and outer diameter measurements using the constant volume law. According to Figure 10, the surface roughness at the die-entrance-side of the drawn tube was smaller than that of the uniaxial tensile-deformed tube, because the *R*_a_ was smaller than that of the uniaxial tensile-deformed tube. However, the *R*_a_ at the die-exit-side was greater than that of the uniaxial tensile-deformed tube. The *R*_a_ was smaller than that of the uniaxial tensile-deformed tube at the die-entrance-side and larger than that of the uniaxial tensile-deformed tube at the die-exit-side.

### 3.3. Quantitative Unevenness Evaluation

**Figure 11:**
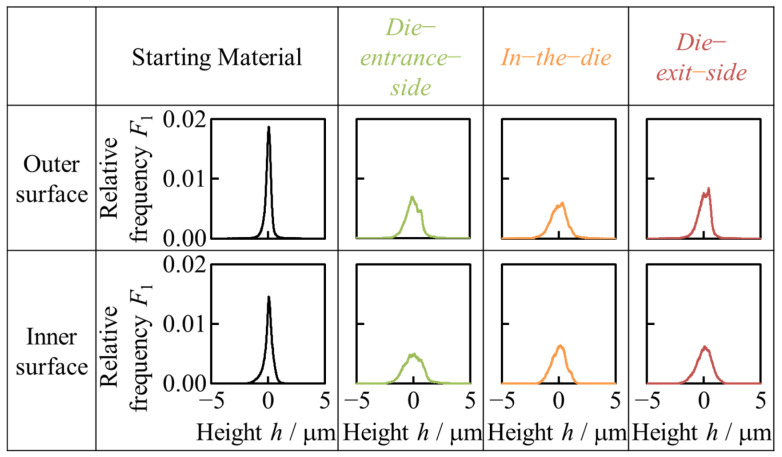
Relative frequency distributions of the height of the starting material and drawn tubes. The relative frequency *F*_1_ was calculated by dividing the frequency at a certain height by the total number of pixels based on the height in Figure 8.

Figure 11 shows the relative frequency distributions of the heights of the starting material and halfway-drawn tubes. The relative frequency *F*_1_ was calculated by dividing the frequency at a certain height by the total number of pixels based on the height in Figure 8. In this study, the starting material had very small surface roughness because there was almost spread in the height distribution and the height concentrated around 0 μm. In contrast, the height distributions of each surface after hollow sinking had two peaks due to the formation of unevenness and height greater than 0 μm on the positive and negative sides, respectively. Therefore, the fitting in the height distribution of the drawn tube is assumed to be a distribution with two peaks. The peak in the relative frequency distribution of height decreased, and the peak became broader from the die entrance side, quantitatively indicating that the inner and outer surface roughnesses developed from the die entrance side. According to Figure 11, there was no significant difference in the height distribution of in-the-die. This indicated that the increase in surface roughness of the inner surface was suppressed as the outer surface was smoothed. The results revealed that smoothing of the outer surface was also important for the smoothing of the inner surface, which is difficult to grind later.

### 3.4. Observation Results in Cross-Section in the Circumferential Direction and the Wall Thickness Direction

**Figure 12:**
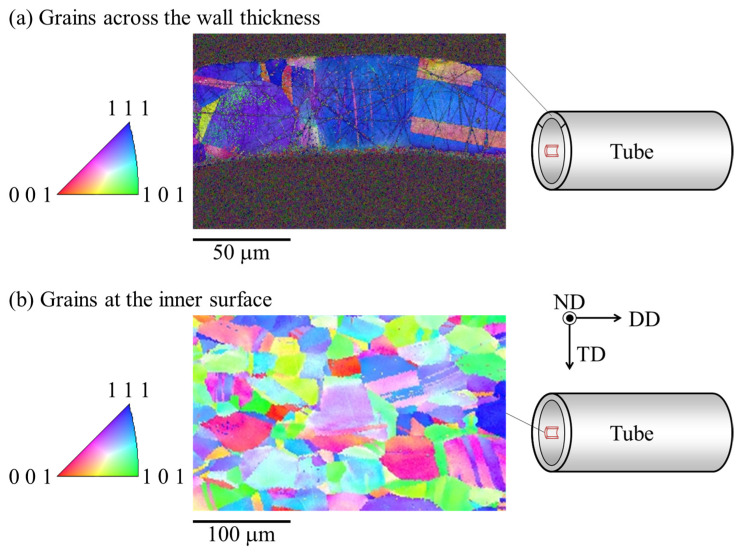
The IPF map of the drawn tube (**a**) across the wall thickness and (**b**) at the inner surface.

**Figure 13:**
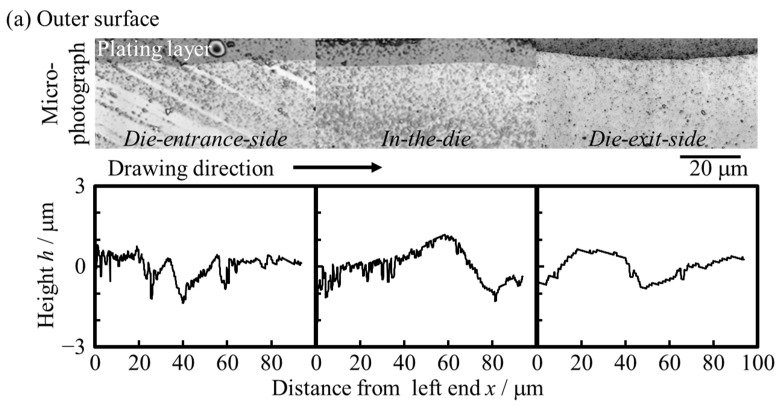

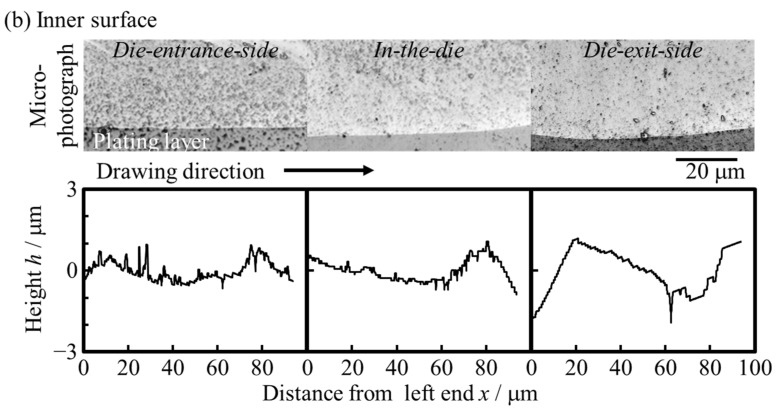
Unevenness calculated from the image of cross-section in the wall thickness direction. The vertical axis represents the variation from the average of the *y*-coordinates of the outer and inner surfaces calculated. The horizontal axis represents the distance from the left edge of the image in Figure 13.

Figure 12 shows the results of EBSD. According to Figure 12, the average grain size (ECD) was larger than the wall thickness of 45 μm. Figure 13 shows the results of the cross-sectional observations in the thickness direction and the calculated unevenness of the inner and outer surfaces. The upper and lower panels represent the outer and inner surfaces, respectively. The observed specimens were divided into two layers: the dark and light layers were plated and a halfway-drawn tube, respectively. It was found that the wall thickness was not constant but rather increased and decreased locally. The image was corrected for tilt using MATLAB^TM^ software. The *y*-coordinates of the upper (outer surface) and lower (inner surface) surfaces were obtained by detecting edges. The variations in the average *y*-coordinates of the outer and inner surfaces were calculated.

## 4. Discussion

### 4.1. Relationship between the Roughness of the Inner and Outer Surface

**Figure 14:**
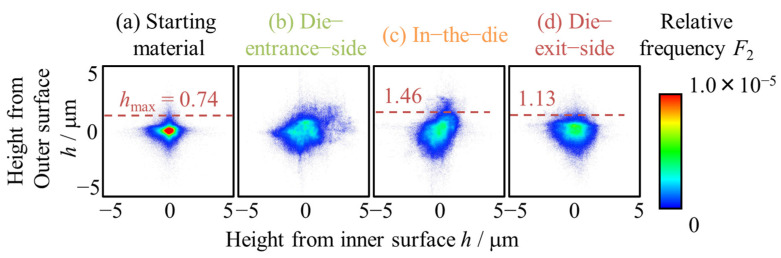
Correlation between the heights of the inner and outer surfaces of the (**a**) starting material (**b**) die-entrance-side, (**c**) in-the-die, and (**d**) die-exit-side of the halfway-drawn tubes. The color bar shows the relative frequency *F*_2_ of height.

The correlation between the heights of the inner and outer surfaces was evaluated based on the height maps of the inner and outer surfaces of the starting material, halfway-drawn tube, and uniaxial tensile deformed, as shown in Figure 14. The relative frequency *F*_2_ was calculated by dividing the frequency at a certain height by the total number of pixels based on the height in Figure 8. In Figure 14, the distribution had a value parallel to the horizontal axis, which indicated that the outer surface was unlikely to exceed a certain value. This indicates that a maximum height exists in the die. This maximum value is defined as the height of the outer surface when the relative frequency is 0.01 of the total height and is called the maximum height *h*_max_. The *h*_max_ at the die-entrance-side, before the contact with the die, was not observed. From Figure 14, it cannot be determined that there is a direct correlation between the heights on the inner and outer surfaces. However, the distribution is similar in shape for the inner and outer surfaces, with a high density in the center and an isotropic decrease in density.

**Figure 15:**
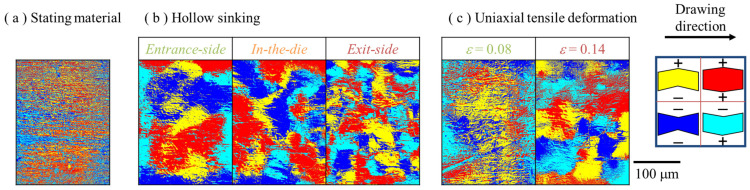
Correlation distribution of inner and outer surface on (**a**) starting material and drawn tube, (**b**) hollow sinking, and (**c**) uniaxial tensile deformation. The correlations of hollow sinking are divided into die-entrance-side, in-the-die, and die-exit-side. The red area indicates convex on both the inside and outside surfaces, the yellow area indicates convex on the outside and concave on the inside, the blue area indicates concave on both the inside and outside surfaces, and the light blue area indicates concave on the outside and convex on the inside.

In this study, the concave and convex shapes indicate positive and negative heights, respectively. The concave–convex relationship was evaluated based on the height distributions in Figure 8 and Figure 10. Figure 15 shows the relationship between the concave and convex surfaces of the inner and outer surfaces of the starting material, halfway-drawn tubes, and uniaxial tensile deformed tubes. Figure 15 evaluates the relationship between the unevenness of the inner and outer surfaces. Positive and negative signs indicate convex and concave shape, respectively, at the height in Figure 8 and Figure 10. The red area indicates convexity on both the inner and outer surfaces; the yellow area indicates convexity on the outer surface and concavity on the inner surface; the blue area indicates convexity on both the inner and outer surfaces; and the light blue area indicates convexity on the outer surface and convexity on the inner surface. When an area is red or blue, the area is thinner or thicker than the average, respectively. These regions were considered areas of grain stretching or shrinkage, given that only one grain was included in the wall thickness. However, the yellow and light blue area correlations were mainly due to grain uplift and downsizing. However, the results of this study did not consider the deformation of the grains themselves; therefore, the transition between grain deformation and unevenness is a subject for future studies.

A correlation of unevenness was randomly formed in the starting material, whereas a correlation of unevenness was formed with a size of area larger than the grain size (approximately 45 μm) after the die entrance side. At the die-entrance-side, only the back tension acts on the tube, which is similar to uniaxial tension. From the die entrance to the die, the area represented by red or blue and the area represented by convex or concave on both the inner and outer surfaces increased. From the in-the-die to the die-exit-side, the correlated distribution ratio of the convex and concave sides approached the uniaxial tensile deformation of the tube.

### 4.2. Transition of Outer and Inner Surface Roughnesses during Hollow Sinking

**Figure 16:**
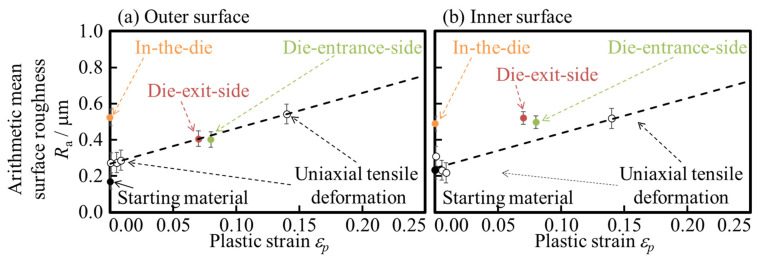
Relationship between the arithmetic surface roughness *R*_a_ and the true strain *ε*_p_ at (**a**) outer surface and (**b**) inner surface, respectively. The black plots represent the die-entrance-side, and the white plots represent uniaxial tension, respectively.

Conventionally, for sheets, the *R*_a_ is proportional to the plastic strain, *ε_p_*, as expressed in Equation (4). *R*_0_ is the initial surface roughness, *d* is the average grain size, and *c* is a constant that depends on the material microstructure. The average grain size was set to *d* = 45 μm, and the grain size was assumed to be one across the wall thickness. The relationship between the surface roughness *R*_a_ and plastic strain *ε*_p_ on the inner and outer surfaces of the uniaxial tensile deformed tubes and drawn tubes is shown in Figure 16. The amount of plastic strain in the drawing direction is 0.08 and 0.14, with an initial length of 100 mm. These values are based on the results of the inner and outer diameter measurements described below based on the constant volume law described in 2.4 Observation of cross-section. The material constant *c* of Figure 16 *c* in this study was 0.02 for both (a) outer surface and (b) inner surface. Additional uniaxial tensile tests were conducted to determine whether uniaxial tensile stress would result in the formation of unevenness on the die-entrance-side. The results were proportional to the strain on both the inner and outer surfaces. Therefore, surface roughness was formed at the entrance of the drawing die by uniaxial tensile deformation. The true strain *ε* of the die entrance was obtained by a constant volume with low inner and outer diameters. The plastic strain *ε*_p_ of the uniaxial tensile deformation was obtained from the strain after unloading in Figure 4 in Section 3.1.

*R*_a_ = *R*_0_ + *c*⋯*d*⋯*ε*_p_ (4)

**Figure 17:**
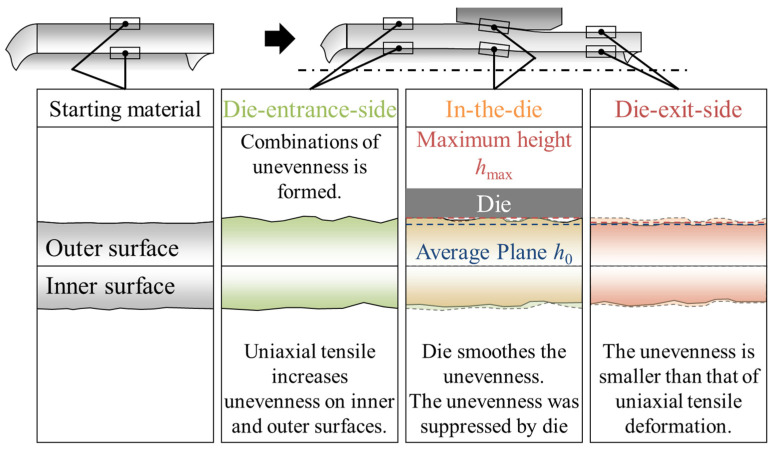
Whole picture of the transitions of inner and outer surface roughnesses during the hollow sinking of the metal microtube. The dotted line in the in-the-die and die-exit-side indicates the standard plane of the surface.

The experimental results and discussion are summarized, and the transitions in the unevenness of the inner and outer surfaces of the metal microtubes during hollow sinking are discussed. Figure 17 illustrates the transition in height distribution on the inner and outer surfaces of the tube during hollow sinking. On the die-entrance-side, unevenness was formed by the uniaxial tension. The surface roughness of the inner and outer surfaces at the die-entrance-side is expressed by the same empirical equation of surface roughening as for the uniaxial tensile deformation. This implies that the magnitude of the back tension can be used to predict the evolution of the surface roughness in metal microtube fabrication. The region with a constant area represents the combination of convex (concave) and concave (convex) surfaces on the inner and outer surfaces. The area of combination is larger than the average grain size. In-the-die, the die smoothened the convex outer surface, resulting in the maximum height of the outer surface exists. On the die-exit-side, the surface roughness is reduced compared with the uniaxial tensile strength on the inner and outer surfaces. The area representing the combination of convex (concave) and concave (convex) surfaces on the inner and outer surfaces was smaller than the average grain size.

## 5. Conclusions

This study aims to clarify the evolution of surface roughness during the hollow sinking of metal microtubes. We measured and compared the inner and outer surface roughness of the starting material, drawn tube, and uniaxial tensile deformation of the tube. The following findings were obtained by examining the relationship between the surface roughness of the inner and outer surfaces:

(1) The surface roughness increases at the die entrance side on the inner and outer surfaces owing to uniaxial tension. Regarding the combination of convex and concave surfaces between the inner and outer surfaces, the starting material has a random arrangement of area sizes smaller than the grain size. However, the sizes of the most convex and concave parts are larger than the grain size on the die entrance side.
(2) The relative frequency distribution of the heights of the inner and outer surfaces of tube in the die and their correlation show that the outer surface has the maximum height with suppressed convexity.
(3) In the die, the outer surface is smoothed by the contact with the die. The increase in inner surface roughness is also reduced, as there is no difference in the shape of the height distribution on the inner and outer surfaces.
(4) The final height of the unevenness at the die exit side is lower than that in the uniaxial tensile tests with the same amount of strain on both the inner and outer surfaces. The area representing the combination of heights on the inner and outer surfaces is smaller than the grain size at the die exit side.
(5) The inner and outer surface roughnesses at the die-entrance-side is formed by uniaxial tensile deformation. Therefore, the arithmetic mean roughness *R*_a_ of die-entrance-side in hollow sinking is expressed by a linear function of plastic strain *ε*_p_. The empirical formula for surface roughness expressed by a linear function of plastic strain *ε*_p_ used in the uniaxial tensile deformation of the sheet material can be used to predict the surface roughness of hollow sinking.

In this study, the surface roughness transition on the inner and outer surfaces of the drawn tube is clarified. This enables the prediction of the surface roughness on the die entrance and exit side of the hollow sinking using plastic strain ε_p_. However, the mechanism of the surface roughness increase in the die is still unknown. This is an issue for the future investigation. In the future, it is necessary to focus on the inhomogeneous deformation of crystal grains, which is a factor in the formation of surface roughness. As a guideline, the surface roughness transition during drawing is simulated by simulation, and the factors that cause the generation and development of surface roughness are discussed.

## Figures and Tables

**Table 1:** Chemical composition of starting material (mass%) [12].

C	Si	Mn	P	S	Ni	Cr	Fe
0.06	0.49	1.78	0.031	0.002	9.21	18.41	Bal.

## Data Availability

Data are contained within the article.
